# Relationship of a public mental health campaign with health service use and association with symptom management knowledge

**DOI:** 10.1192/bjo.2025.55

**Published:** 2025-05-21

**Authors:** Paul McCrone, Claire Henderson

**Affiliations:** Institute for Lifecourse Development, University of Greenwich, UK; Health Service and Population Research Department, Institute of Psychiatry, Psychology & Neuroscience, King’s College London, UK

**Keywords:** Public mental health, mental health promotion, economic analysis, cost-effectiveness

## Abstract

**Background:**

Mental health literacy can potentially be improved through a public mental health campaign. The aim of the campaign Every Mind Matters (EMM) was to support adults to help address common subclinical mental health problems and improve their mental well-being and literacy, by using its National Health Service-endorsed digital resources.

**Aims:**

Although not an objective of the campaign itself, this study aims to (a) address the relationship of EMM through the use of general practitioners and mental health therapists and (b) explore the association of EMM with symptom management knowledge.

**Method:**

Health Survey for England 2019 data were obtained on campaign awareness, uptake of campaign materials and the use of general practitioners and therapists. Logistic regression models were used to explore the impact of the campaign on whether services were used, and ordered logistic models explored the impact on the number of contacts. Campaign costs were viewed alongside symptom management outcomes.

**Results:**

The analyses included 2023 individuals. Of those campaign aware, 16% had contact with a general practitioner for mental health reasons compared with 9% of those who were campaign unaware. Those who were campaign aware were also significantly more likely to have seen a mental health therapist. The campaign cost per unit improvement in symptom management knowledge was below £20.

**Conclusions:**

Contact with general practitioners and therapists was associated with campaign awareness. If even a small proportion of symptom management knowledge improvement is due to the campaign, then it has the potential to be cost-effective. Further work is required to establish this.

According to the 2014 Adult Psychiatric Morbidity Survey, common mental disorders occur in one in six adults in England.^1^ These disorders include depression, generalised anxiety disorder, panic disorder, phobias, obsessive compulsive disorder and unspecified common disorders. Linked to approaches to treating conditions, there is a recognition that knowledge about mental health is important. Mental Health Literacy (MHL) has been defined as (a) knowledge of how to obtain and maintain positive mental health, (b) knowledge of the symptoms and management of stress, anxiety and low mood, (c) help-seeking self-efficacy and the ability to promote one’s own mental health and (d) stigma related to mental disorders.^2^

Promoting MHL at a population level is seen as an important public mental health goal, due to the expectation that it should result in earlier help-seeking or effective self-management, and hence better population mental health and reduced burden on the healthcare system. A further expectation is that it should promote recognition and more effective response to others’ mental health problems as well as one’s own, and also that it reduce actively harmful responses through stigma reduction. While there is evidence for an effect on both stigma and the confidence to help others, this is mixed,^3^ and evidence for an impact on service use is lacking.

Public Health England (PHE) launched, and gradually enhanced, a web resource backed by a promotional campaign (to lead people to the web resource) called Every Mind Matters (EMM) in October 2019 following pilot work in 2018. This aimed to support adults in taking positive self-care actions regarding their mental health and MHL. EMM incorporated various digital resources to guide people in addressing stress, anxiety, low mood and sleep problems (and so did not target only diagnosed mental health problems). The provision of these resources was aimed at helping prevent mental health conditions from worsening and requiring National Health Service treatment. EMM was piloted in the West Midlands region of England, and was revised for the national launch in October 2019 to emphasise the delivery and promoting of evidence-based digital resources to facilitate self-care, in addition to providing information about conditions. A second campaign ran in January 2020 to drive people to the EMM web resource to encourage self-care action. This was followed by campaigns to promote actions for people to take care of their mental health during the first COVID-19 lockdown, followed by a targeting of parents to support them in looking after their children’s mental health. These latter developments occurred following data collection for this study.

Because public health campaigns use resources that could be deployed for other purposes, it is important to assess their cost-effectiveness. A key consideration is whether we are interested in looking at costs related to campaign exposure, or at those related to campaign effects.^4^ It is also of interest to determine whether the introduction of a campaign has a subsequent impact on the use of health and other services. Policy-makers might even be cautious about rolling out a campaign if it is likely to result in much greater use of services (unless that is a specific objective). Few economic evaluations of health campaigns have been conducted.^5^ We previously conducted evaluations of an anti-stigma campaign;^6,7^ however, that work relied on modelling, and we did not have data on the use of healthcare services associated with campaign uptake.

The EMM campaign itself was not designed to impact healthcare use; however, this is of interest from an economic perspective. The aims of this study therefore were to assess whether (a) campaign awareness and engagement are associated with primary care and other healthcare contacts, and (b) campaign outcomes are sufficient to justify the costs incurred in terms of potential cost-effectiveness.

## Method

### Data sources and variables

This paper is based on secondary analysis of anonymised data from a YouGov survey and from the Health Survey for England (HSE). The study did not require additional ethical approval or consent. Data were obtained from the 2019 HSE.^8^ The HSE began in 1991 and is carried out annually. It is a cross-sectional household survey and, while some core questions are always asked, additional questions are incorporated into each year’s version. In 2019, specific questions relating to the EMM campaign were included in the survey, which went out to 8205 adults. These were ‘Ever seen or heard of Every Mind Matters’ and ‘How many times before HSE interview, looked at Every Mind Matters videos or info’. Data are collected throughout the year, and the quarter in which this happens is recorded. We included data only from the final quarter, because the EMM campaign commenced in October 2019. From the survey we extracted information on demographic characteristics (gender, age, marital status and ethnicity), self-assessed general health (very good/good, fair, bad/very bad) and whether a physical or mental health problem was reported. The EMM campaign was piloted in the West Midlands region of England, and therefore we included a variable that indicated whether respondents were from that area. We also extracted data on whether contact had been made with general practitioners and therapists for mental health reasons (including counsellors and psychotherapists) during the previous year, and the number of these contacts.

Information on the costs of running the EMM campaign was obtained directly from PHE; these were estimated to be £3.5 million over the period covered by the HSE data (PHE, personal communication, 2023).

Effectiveness of the campaign was derived from a repeated cross-sectional survey, conducted by YouGov, which related to mental health awareness and literacy.^9^ Six waves of the survey, with data on campaign awareness, were conducted between September 2019 and March 2021.

For our study, we used information on two specific areas that showed most change and were measured using scales designed specifically with the campaign aims in mind,^9^ which were improvements in knowledge of symptom management of depression and anxiety. These were measured using the Mental Health Literacy – Action (MHL-ACT) scale, with scores ranging from 0 to 7 (higher scores indicate better management knowledge).

### Analysis

The HSE data sample was divided into two groups: those who were campaign unaware and those who were campaign aware. Demographic and clinical characteristics were compared between these two groups. The variables were all categorical, and chi-square tests were used to assess the statistical significance (defined at the *P* < 0.05 level) of group differences. We then repeated this for those who had made use of the campaign materials and those who had not.

Use or not of general practitioners and mental health therapists was reported, with the former divided into use for mental health and use for physical health problems. (It should be noted these were not mutually exclusive.) Use of therapists was not linked to specific reasons (e.g. depression or anxiety). In investigating the relationship between campaign awareness and use of services, we controlled for age and gender using a logistic regression model. A similar model was used to investigate the relationship between the number of times campaign material was accessed and the use or not of general practitioners and therapists.

The number of general practitioner contacts (not subdivided according to reason) was analysed using an ordered logistic model. The levels used for the dependent variable were no contact, 1–2 contacts, 3–5 contacts, 6–10 contacts and over 10 contacts. The no-contact level was used as the reference category with which other levels were compared.

To explore potential cost-effectiveness, we calculated the ratio between campaign cost and improvement in knowledge about symptom management for both depression and anxiety using the YouGov survey data. Economic evaluations require a comparator and the use of incremental cost-effectiveness ratios. In the absence of a comparator, we made assumptions as to how much improvement could be attributed to the campaign itself, and these ranged from 10 to 100%.

## Results

There were 8204 respondents aged 16 years or over included in the HSE sample, of whom 2023 were from the final quarter of the year. The number who were aware of the campaign was 695 (34%) (Table 1). Those who were campaign aware were significantly more likely to be female and relatively young;. There were small but statistically significant differences between these groups in regard to ethnicity and self-assessed general health; those who were campaign aware were significantly more likely to mention that they had a mental health problem; those who had made use of the campaign were also more likely to be female and relatively young than those who did not, and they were also more likely to have self-reported mental health problems.

**Table 1:** Characteristics of sample, by campaign awareness and use

Characteristic	Campaign unaware (*n* = 1328)	Campaign aware (*n* = 695)	Chi^2^ *P*-value	No use of campaign (*n* = 1692)	Some use of campaign (*n* = 329)	Chi^2^ *P*-value
	*n* (%)	*n* (%)		*n* (%)	*n* (%)	
Female sex	660 (49.7)	429 (61.7)	<0.001	878 (51.2)	209 (63.5)	<0.001
Age (years)			<0.001			<0.001
16–24	113 (8.5)	63 (9.1)		142 (8.4)	34 (10.3)	
25–34	135 (10.2)	137 (19.7)		198 (11.7)	74 (22.5)	
35–44	212 (16.0)	152 (21.9)		296 (17.5)	68 (20.7)	
45–54	208 (15.7)	134 (19.3)		274 (16.2)	68 (20.7)	
55–64	222 (16.7)	126 (18.1)		294 (17.4)	53 (16.1)	
65–74	239 (18.0)	63 (9.1)		274 (16.2)	28 (8.5)	
75+	199 (15.0)	20 (2.9)		214 (12.7)	4 (1.2)	
Marital status/living arrangements			<0.001			<0.001
Single	232 (17.5)	135 (19.5)		291 (17.2)	76 (23.1)	
Married	720 (54.2)	348 (50.1)		915 (54.1)	152 (46.2)	
Separated	21 (1.6)	18 (2.6)		26 (1.5)	13 (4.0)	
Divorced	112 (8.4)	43 (6.2)		135 (8.0)	20 (6.1)	
Widowed	116 (8.7)	21 (3.0)		127 (7.5)	9 (2.7)	
Cohabiting	127 (9.6)	129 (18.6)		197 (11.7)	59 (17.9)	
White ethnicity	1092 (82.7)	611 (87.9)	0.002	1415 (84.0)	286 (86.9)	0.183
Living in West Midlands	123 (9.3)	78 (11.2)	0.175	159 (9.4)	42 (12.8)	0.062
Self-assessed general health			0.031			0.681
Very good/good	962 (72.5)	539 (77.6)		1259 (74.5)	241 (73.3)	
Fair	249 (18.8)	113 (16.3)		297 (17.6)	64 (19.5)	
Bad/very bad	116 (8.7)	43 (6.2)		135 (8.0)	24 (7.3)	
Self-reported health problem	600 (45.3)	273 (39.4)	0.011	731 (43.3)	140 (42.6)	0.808
Self-reported mental health problem	92 (6.9)	79 (11.4)	0.001	127 (7.5)	44 (13.4)	<0.001

### Use of general practitioners and therapists

Similar proportions of those who were campaign aware and unaware reported some contact with general practitioners for physical health reasons, and significantly more had contact for mental health reasons (Table 2). (It should be noted that these categories are not mutually exclusive.) However, the number of contacts with general practitioners did not differ significantly. There was a significantly higher likelihood that those who were campaign aware would have had contact with a therapist, although the numbers were relatively low. Across all those included, 7.2% had therapist contacts. The most common types of therapy were counselling (2.1%), cognitive behavioural therapy (2.0%) and psychotherapy/psychoanalysis (1.8%).

**Table 2:** Number (%) of sample with general practitioner and therapist contacts

Service	Campaign unaware (*n* = 1328)	Campaign aware (*n* = 695)	Chi^2^ *P*-value	No use of campaign (*n* = 1692)	Some use of campaign (*n* = 329)	Chi^2^ *P*-value
Any contact with general practitioner for physical health reasons	960 (73.7)	482 (70.2)	0.090	1202 (72.3)	238 (73.2)	0.737
Any contact with general practitioner for mental health reasons	117 (9.0)	109 (15.9)	<0.001	166 (10.0)	60 (18.5)	<0.001
Number of general practitioner contacts			0.885			0.263
0	304 (23.4)	162 (23.6)		403 (24.3)	63 (19.4)	
1–2	490 (37.6)	269 (39.2)		621 (37.4)	137 (42.2)	
3–5	312 (24.0)	164 (23.9)		400 (24.1)	75 (23.1)	
6–10	123 (9.5)	57 (8.3)		151 (9.1)	29 (8.9)	
10+	73 (5.6)	35 (5.1)		87 (5.2)	21 (6.5)	
Any contact with therapist	76 (5.7)	70 (10.1)	<0.001	107 (6.3)	39 (11.9)	<0.001

After controlling for background variables, being campaign aware was seen to be significantly associated with contacting a general practitioner for mental health reasons (Table 3); it was not significantly associated with contact for physical health reasons or contacting a therapist. Being in the oldest age category was significantly associated with greater use of general practitioners for physical health reasons, but with less use for mental health reasons and the use of therapists. Female gender was significantly associated with greater use of all services. Those of White ethnicity were less likely to see a general practitioner for physical health reasons than were Black and minority ethnic respondents. Compared with very good/good health, being in fair health or bad/very bad health was associated with greater use of all services. There was no separate impact of residing in the West Midlands.

**Table 3:** Relationship between campaign awareness and contact with general practitioners and therapists

	Contact with general practitioners for physical health reasons		Contact with general practitioners for mental health reasons		Contact with therapists	
	OR	CI	OR	CI	OR	CI
Campaign awareness	0.98	0.78–1.22	1.57	1.14–2.15	1.37	0.95–1.99
Age (years)^a^						
25–34	0.86	0.54–1.35	1.73	0.93–3.24	1.36	0.66–2.82
35–44	0.82	0.52–1.29	2.14	1.15–3.98	1.49	0.72–3.08
45–54	1.06	0.66–1.69	0.95	0.48–1.87	0.95	0.44–2.06
55–64	1.20	0.73–1.96	0.93	0.47–1.87	0.61	0.27–1.38
65–74	1.61	0.96–2.71	0.29	0.12–0.70	0.15	0.05–0.48
75+	2.63	1.38–4.98	0.22	0.08–0.60	0.16	0.05–0.56
Female	1.92	1.55–2.36	1.44	1.05–1.98	1.61	1.10–2.34
Marital status^b^						
Married	1.63	1.18–2.25	0.34	0.22–0.51	0.51	0.31–0.84
Separated	1.66	0.73–3.76	0.82	0.33–2.06	1.47	0.57–3.84
Divorced	1.35	0.82–2.21	0.97	0.53–1.76	1.15	0.57–2.31
Widowed	1.12	0.61–2.06	0.57	0.22–1.46	0.64	0.18–2.26
Cohabiting	1.25	0.86–1.82	0.46	0.27–0.77	0.42	0.22–0.80
White ethnicity	0.64	0.47–0.87	1.59	1.00–2.52	2.62	1.43–4.82
Living in West Midlands	0.72	0.51–1.01	1.47	0.90–2.41	1.34	0.74–2.42
Self-assessed health^c^						
Fair	1.80	1.33–2.45	2.99	2.03–4.43	2.49	1.56–3.97
Bad/very bad	3.09	1.84–5.20	11.10	7.09–17.37	6.66	4.01–11.06
Constant	1.57		0.06	0.03–0.11	0.03	

OR, odds ratio.

a Reference category: under 25 years.

b Reference category: single.

c Reference category: very good/good health.

The number of general practitioner contacts was not significantly associated with campaign awareness (Table 4). This number was significantly higher for those who were older, women, those who were Black and minority ethnic and those with health that was fair or bad/very/bad (compared with good health).

**Table 4:** Relationship between campaign awareness and number of general practitioner contacts

	Contact with general practitioners for physical health reasons	
	OR	CI
Campaign awareness	1.08	0.91–1.29
Age (years)^a^		
25–34	1.32	0.89–1.94
35–44	1.10	0.75–1.61
45–54	1.30	0.87–1.93
55–64	1.16	0.77–1.75
65–74	1.61	1.06–2.46
75+	1.96	1.22–3.16
Female	1.70	1.43–2.00
Marital status^b^		
Married	1.20	0.92–1.57
Separated	1.33	0.72–2.48
Divorced	1.20	0.81–1.77
Widowed	1.08	0.70–1.69
Cohabiting	1.13	0.82–1.56
White ethnicity	0.62	0.49–0.79
Living in West Midlands	1.00	0.76–1.32
Self-assessed health^c^		
Fair	2.99	2.38–3.75
Bad/very bad	11.30	8.10–15.76

OR, odds ratio.

a Reference category: under 25 years.

b Reference category: single.

c Reference category: very good/good health.

The number of times that people actually made use of the EMM campaign was not significantly associated with use or not of general practitioners for physical health reasons, or of therapists (Table 5). Those who used EMM resources three to five times were more likely to have seen a general practitioner for mental health reasons compared with those with no use of the campaign.

**Table 5:** Relationship between campaign uptake and use of general practitioner services and therapists

	Contact with general practitioners for physical health reasons		Contact with general practitioners for mental health reasons		Contact with therapists	
	OR	CI	OR	CI	OR	CI
EMM use^a^						
1 time	1.20	0.77–1.86	1.22	0.68–2.22	1.58	0.85–2.97
2 times	1.27	0.74–2.16	1.41	0.75–2.64	1.19	0.57–2.48
3–5 times	1.46	0.76–2.80	2.09	1.00–4.37	0.69	0.20–2.33
6+ times	0.94	0.55–1.61	1.55	0.79–3.04	1.57	0.75–3.29
Age (years)^b^						
25–34	0.85	0.54–1.35	1.74	0.93–3.26	1.34	0.65–2.80
35–44	0.84	0.53–1.32	2.18	1.16–4.08	1.48	0.71–3.08
45–54	1.08	0.68–1.74	0.95	0.48–1.89	0.92	0.42–2.00
55–64	1.22	0.75–2.01	0.93	0.46–1.88	0.60	0.26–1.37
65–74	1.67	1.00–2.81	0.27	0.11–0.67	0.14	0.04–0.46
75+	2.77	1.46–5.25	0.21	0.07–0.57	0.15	0.04–0.53
Female	1.90	1.54–2.34	1.45	1.05–1.99	1.62	1.11–2.37
Marital status^c^						
Married	1.63	1.18–2.25	0.34	0.22–0.52	0.52	0.31–0.85
Separated	1.62	0.71–3.69	0.84	0.34–2.09	1.40	0.53–3.65
Divorced	1.35	0.82–2.22	0.98	0.54–1.79	1.16	0.57–2.34
Widowed	1.12	0.61–2.06	0.57	0.22–1.47	0.65	0.19–2.27
Cohabiting	1.23	0.85–1.79	0.48	0.29–0.81	0.43	0.23–0.83
White ethnicity	0.63	0.46–0.86	1.65	1.04–2.60	2.72	1.48–5.00
Living in West Midlands	0.70	0.50–0.99	1.43	0.87–2.35	1.39	0.76–2.53
Self-assessed health^d^						
Fair	1.78	1.31–2.42	2.96	2.00–4.38	2.46	1.54–3.95
Bad/very bad	3.07	1.82–5.17	10.87	6.93–17.05	6.56	3.92–11.00
Constant	1.52		0.06	0.03–0.12	0.03	

OR, odds ratio; EMM, Every Mind Matters.

a Reference category: no use of EMM.

b Reference category: under 25 years.

c Reference category: single.

d Reference category: very good/good health.

If respondents had used EMM resources three to five times, they were significantly more likely to have had more general practitioner contacts compared with those not having used the resources at all (Table 6). However, this did not apply to other levels of EMM use.

**Table 6:** Relationship between campaign uptake and use of general practitioner services and therapists

	OR	CI
EMM use^a^		
1 time	1.15	0.81–1.64
2 times	1.02	0.69–1.51
3–5 times	1.64	1.00–2.69
6+ times	1.09	0.70–1.69
Age (years)^b^		
25–34	1.30	0.88–1.93
35–44	1.10	0.75–1.63
45–54	1.30	0.87–1.94
55–64	1.15	0.76–1.74
65–74	1.60	1.05–2.45
75+	1.97	1.22–3.18
Female	1.69	1.43–2.00
Marital status^c^		
Married	1.21	0.92–1.58
Separated	1.32	0.71–2.47
Divorced	1.21	0.82–1.78
Widowed	1.10	0.71–1.71
Cohabiting	1.14	0.82–1.57
White ethnicity	0.62	0.49–0.79
Living in West Midlands	1.00	0.76–1.31
Self-assessed health^d^		
Fair	3.00	2.39–3.77
Bad/very bad	11.45	8.20–15.99

OR, odds ratio; EMM, Every Mind Matters.

a Reference category: no use of EMM.

b Reference category: under 25 years.

c Reference category: single.

d Reference category: very good/good health.

### Relationship between costs and campaign effectiveness

The total cost of the EMM campaign was estimated to be £3.5 million over the HSE data collection period. Elsewhere, it is reported from the YouGov survey that, of 13 178 respondents across six waves, 33.6% were aware of the campaign, with awarenerss in individual waves ranging from 18.1 to 43.2%.^10^ If this figure of 33.6% is representative of the adult population, it equates to 14.8 million people based on UK census data. The cost therefore for each person being campaign aware is £0.24. (The range would be £0.13–0.31 based on the alternative awareness figures.) From the survey, we also know that the benefits in terms of knowledge about symptom management of anxiety and depression were improvements of 0.18 and 0.13 units, respectively, on the scale of 0–7. If all of this benefit is attributable to the campaign, the cost per unit improvement for knowledge of symptom management is £1.31 and £1.82 and for anxiety and depression, respectively; if only 10% is due to the campaign, these figures are £13 and £18, respectively.

## Discussion

There have been only a small number of economic studies of population-level mental health campaigns.^6,7^ We found that being EMM campaign aware was associated with higher contact with general practitioners for mental health reasons. Awareness was not associated with the number of general practitioner contacts, and the extent to which campaign materials were used was not significantly associated with service use. The increased rate of contact could be explained by the Health Belief Model, whereby uptake of care is linked to perceived level of health problems,^11^ especially because this perception is influenced by exposure to campaigns such as EMM. Alternatively, if individuals are open to social influences such as greater awareness brought about by EMM, these influences may affect behaviour such as seeking support, as suggested by social cognitive theory.^12^

Preliminary analyses of the association between costs and outcomes have been made, and these suggest that the campaign can potentially achieve positive outcomes in terms of mental health literacy at a modest cost. Elsewhere, the findings of Evans-Lacko et al in their evaluation of the ‘Time to Change’ anti-stigma marketing campaign reveal that the costs per person with improved attitudes to mental health were extremely low.^7^ Large public mental health campaigns are relatively rare and have to compete for funds. Investment in such promotional and preventative initiatives may not be seen as attractive as funding treatment for those who have developed specific conditions. They may also struggle due to the inevitably limited evidence base. Evaluating campaigns is challenging given that they do not align easily with conventional evaluative approaches such as randomised trials.

### Limitations

HSE data collection was reduced due to the COVID-19 pandemic. Although this meant that data from only 3 months of the HSE were used, these still amounted to reasonably high numbers.

In terms of the relationship between campaign awareness and use of services, we are not able to determine cause and effect. While this is a limitation, it is actually likely to be bidirectional because those with mental health concerns may be more likely to recognise the campaign, but it may also encourage them to seek help. We were constrained by the time periods over which the campaign was delivered and the HSE data were collected. However, investigating the impact of a campaign such as EMM in service use is important, and a future study should be designed to explicitly address this by collecting data relating to sequential non-overlapping time periods.

The investigation of potential cost-effectiveness was limited in that we do not have any comparator. We did have an estimate of the campaign cost, but it is unclear what the denominator should be. We chose to use the number of people who would potentially be exposed to the campaign. Without a comparison group we cannot know how much improvement in anxiety and depression symptom management knowledge is due to the campaign, and we therefore used a range of estimates. While this is informative, a more robust form of comparison would allow greater confidence in directing policy.

Furthermore, the outcome – knowledge about symptom management – has limitations. It may result in actual changes about such management, but that is not guaranteed. Again, the Health Belief Model suggests that increased awareness may result in help-seeking behaviour as a way of better managing conditions.

Finally, data were obtained from a variety of sources. This was required for the analyses, but combining information from disparate sources is challenging. In future analyses of such campaigns, the requirement for appropriate data collection and linkage needs to be emphasised and, ideally, prospective data should be obtained.

The study has shown that being aware of a mass-marketing campaign around mental health awareness is associated with increased contact with some services for mental health reasons. This relationship needs to be further explored to establish the causal direction and, in future research studies, direct measurement of service use and engagement with campaigns should be enabled. We cannot yet say that a campaign like this is cost-effective, but indications are that costs are low in relation to potential benefits.

### Lived experience commentary by Lizzie Mitchell and Andrew Grundy

A previous Lived Experience Commentary (in Stuart et al^13^) has highlighted some of the limitations of EMM, in that it excludes people with a pre-‘diagnosed’ mental health condition, those whose first language is not English and those with digital accessibility difficulties. This paper highlights that the campaign is not encouraging males or older adults to seek help, compared with females (trans and non-binary not reported). Whatever good EMM is achieving in terms of awareness and help-seeking (and at a low cost), it is not for ‘every mind’.

EMM increased self-awareness of mental health conditions, and self-efficacy in managing emotions. However, recent public mental health campaigns have focused on increasing personal responsibility, consequently shifting away from holding the government responsible for meeting the urgent demand for mental health services. With therapy being the National Institute for Health and Care Excellence-recommended treatment for common mental health problems, this paper suggests that barriers were still faced in accessing treatment from general practitioners. Without increased service provision, increasing MHL is inadequate in regard to addressing rising mental health problems.

The government should consider investing in tracking the reach of campaigns more accurately, and in implementing better feedback systems. Consequently, more accurate numbers would help evidence these campaigns’ effectiveness and cost-effectiveness, rather than relying on estimates. The paper highlights EMM’s value over a 3-year period, but we are concerned about the longer-term plans for public health campaigns. Current plans are unclear, and we would like clarity on how the government plans to address MHL within an increasingly needy population.

Lived experience involvement was lacking in this study, and we advocate for lived experience involvement in the design, conduct and reporting of health economic evaluation studies.

## Data Availability

Data were provided by YouGov and the Office for National Statistics. Applications for access to the data should be made to these bodies.
